# School and Community Stakeholder Perceptions of a Free, Confidential Digital Mental Health Platform (Soluna): Mixed Methods Study Examining Barriers and Facilitators to Real-World Implementation at Scale and Early Impact

**DOI:** 10.2196/82864

**Published:** 2026-03-04

**Authors:** Jacqlyn Yourell, Jennifer Huberty, Regina Misch, Louisa Salhi

**Affiliations:** 1 Fit Minded Inc Phoenix, AZ United States; 2 Kooth Digital Health Chicago, IL United States; 3 Threshold LLC York, SC United States; 4 Kooth Digital Health London, England United Kingdom; 5 University of Manchester Manchester, England United Kingdom

**Keywords:** adolescent mental health, digital mental health, mobile health, app, implementation science, stakeholder perspectives, schools, community organizations

## Abstract

**Background:**

Mental health disorders are common among adolescents worldwide; yet, access to preventive and early intervention services remains limited. Digital mental health platforms may help bridge this gap, but little is known about how these platforms are perceived, implemented, and adopted by school and community stakeholders during early stages of rollout in real-world youth-serving settings.

**Objective:**

This study aimed to examine school and community stakeholders’ perceptions of a new, free, confidential digital mental health platform for youth (Soluna by Kooth Digital Health) in its first year of rollout, focusing on its features, barriers, and facilitators to early implementation and adoption, and early stakeholder perceptions of its perceived impact on youth mental health.

**Methods:**

Surveys were distributed to 77 stakeholders (54 school staff and 23 community staff) in California from February to April 2025. Eligible participants were frontline staff directly engaging with youth in settings where the platform was offered. Following the survey, 17 stakeholders (12 school and 5 community) participated in semistructured interviews via Zoom (Zoom Communications). Survey data were analyzed descriptively to summarize perceptions and experiences, while interview data were analyzed using inductive-deductive reflexive thematic analysis to explore themes related to implementation, facilitators, and barriers.

**Results:**

Most stakeholders (71.4%) agreed that the platform positively contributed to youth well-being, and 89.6% felt comfortable referring youth to it, indicating good acceptability. Free access (91%), availability during nontraditional hours (45%), and ease of use (42%) were identified by stakeholders as the most valuable features for youth. Facilitators of adoption included digital accessibility (87%), confidential peer support (84.1%), and youth choice in engagement (85.7%). Key barriers included stigma around mental health (64.7%) and lack of awareness of the platform’s benefits (61%). Qualitative findings revealed three main categories: (1) perceived impact, including support for youth from diverse backgrounds and use as a supplemental resource when traditional services are limited; (2) facilitators to implementation and adoption, such as direct referrals, peer promotion, accessible framing focused on coping and life skills, supportive materials, and engagement from platform staff; and (3) barriers to implementation and adoption, including stigma and phone or internet access challenges in school settings. Stakeholders also emphasized the importance of practical resources and ongoing support to build confidence in using and recommending the platform.

**Conclusions:**

Stakeholders viewed the platform as a valuable and accessible tool to support youth mental health, particularly in underserved communities. Findings suggest that digital mental health platforms should address external barriers to adoption, including stigma around mental health, while also increasing awareness of available resources. Providing tailored implementation support demonstrates progress in these areas and can further strengthen adoption and engagement. These findings offer actionable recommendations for improving the design and delivery of digital mental health platforms in real-world youth-serving settings.

## Introduction

Mental health disorders affect 15.2% of children and adolescents aged 10-19 years globally, with anxiety disorders most prevalent (4.9%), followed by depressive disorders (2.4%) [1]. In the United States, rates are substantially higher, with 31.9% of adolescents aged 13-18 years experiencing anxiety disorders and 14.3% experiencing mood disorders [2]. These disorders contribute to significant morbidity and mortality, with suicide ranking as the second leading cause of death among adolescents aged 15-19 years [3]. Multiple risk factors, including untreated mental disorders, family dysfunction, and acute psychosocial stressors, interact in complex ways to increase vulnerability, making early prevention efforts critical [3]. However, accessing preventive and early intervention services remains challenging. Shortages of licensed mental health care professionals, long wait times, and financial barriers limit timely access to care [4]. These challenges are especially pronounced in geographically underserved areas, such as rural communities, where psychological support may be scarce or entirely unavailable [5].

Adolescents and young adults are turning to mobile apps as a means of supporting their mental health and wellness more frequently [6]. However, many existing digital mental health services are accessible only through paid subscriptions or insurance-based models [7]. Insurance-based models in digital mental health services are those that can only be accessed when the cost is covered through a formal health care system, meaning individuals must qualify for coverage through their country’s health services or employment benefits rather than being able to access the service freely and independently. These financial hurdles can lead to lower user engagement; adolescents and young adults report that they expect mental health apps to be free and are often reluctant to download apps that require payment when free alternatives are available [8-11].

The stigma surrounding mental health can deter adolescents from seeking help through traditional channels. For example, traditional mental health services (eg, outpatient clinics or private therapy sessions) may seem intimidating and overly clinical to adolescents, while parent involvement can amplify concerns about privacy and stigma [12]. Given that schools and community programs are central to how young people learn about and engage with mental health and wellness services [12-14], it is essential to provide mental health tools through these accessible, familiar avenues. In addition, digital mental health platforms that offer round-the-clock access to mental health support are crucial for those who face barriers to accessing face-to-face services (eg, geographically diverse areas and areas with shortages of licensed therapists) [5,15].

Given the rapid expansion of the digital health marketplace, now comprising over 350,000 mobile health apps worldwide [16], it is important to explore which aspects of real-world implementation are successful and scalable to ensure these platforms are sustained. In this context, a platform is defined as a multicomponent digital mental health system that integrates multiple services within a single digital environment, which may be accessed via a mobile app. Insights from established companies with successful platform rollouts can help identify key barriers and facilitators of scalable implementation, informing the launch of new platforms across diverse regions. Criteria for a successful rollout include effective adoption and sustained use across settings, consistent with implementation science frameworks [17]. Exploring this process in the first year of a platform’s rollout provides an opportunity to capture early stakeholder-identified impacts, including potential systemic ripple effects that may not be easily quantifiable in broader health or education datasets.

The purpose of this paper is to provide a nuanced understanding of how school and community stakeholders view the offering of a free, confidential digital mental health platform for youth in their schools and communities during its first year of rollout. Guided by implementation science frameworks, including the Consolidated Framework for Implementation Research (CFIR) and Reach, Effectiveness, Adoption, Implementation, and Maintenance (RE-AIM) frameworks [18,19], this mixed methods study addresses the following aims:

1. Examine stakeholders’ views of the platform’s perceived impact on youth mental health and well-being as well as school or organizational contexts;
2. Identify barriers and facilitators influencing early implementation and adoption in real-world school and community settings; and
3. Explore downstream or distal effects of platform implementation, including perceived changes in staff workload and youth engagement in school and community settings.

## Methods

**Overview**

Throughout this paper, adolescents and young adults are collectively referred to as “youth.” This terminology is used because the platform is available to those aged 13-25 years, and stakeholders in school and community settings provided general observations about youth in their settings, rather than reporting on specific individual users.

### Soluna Platform

Kooth Digital Health has a longstanding history of scaled implementation in education and health care settings across the United Kingdom [20]. Building on this experience, the Soluna platform was launched by Kooth Digital Health, a commercial digital mental health company, at the start of 2024 [21]. Soluna is a free, confidential digital mental health and well-being platform designed to expand access to preventive and early intervention support for youth aged 13-25 years. Accounts can only be created by individuals within this age range, although demonstration versions are available for training and outreach purposes. The rollout of Soluna applies implementation processes used in previous settings to a new geographic context (the United States), creating an opportunity to examine early barriers, facilitators, and perceived impacts as observed by frontline stakeholders in youth-serving settings.

During Soluna’s rollout, the platform was, and at the time of writing remains, available to all youth (aged 13-25 years) across California in a population-level approach [22-24], at no cost and without referral, insurance, diagnosis, immigration disclosure, or parental consent. Youth learned about Soluna through routine outreach and promotion efforts, including schools, community-based organizations, in-person events, digital media, and word of mouth. Youth could access Soluna by downloading the mobile app and independently registering using either a guest or a full account. Guest registration provided access to self-guided tools and content, while full registration enabled access to moderated peer support, one-to-one coaching with trained professionals, and care navigation services. Soluna is deployed statewide as part of the California Youth Behavioral Health Initiative [25]. This initiative aims to expand access to mental health services for youth throughout the state, particularly in underserved communities, and the Soluna platform plays a key role in supporting the California Youth Behavioral Health Initiative’s goal of providing accessible, scalable mental health resources to California’s youth population [26,27].

Soluna is free, eliminating financial and administrative barriers to participation and ensuring that young people can access routine mental health support anytime and anywhere. Users can search a repository for local clinical and nonclinical support, while the contact center provides translation services and technical assistance. The platform enables personalized, one-to-one support with trained professionals through live chat, telephone, or video sessions and offers coaching either on a drop-in basis or by scheduled appointment. In addition, users can journal, set and track progress on goals, and contribute to a human-moderated peer community to ensure safety and content appropriateness. The Soluna platform also includes robust care navigation services; highly trained care coordinators assist users in locating resources beyond the platform, such as services providing food, housing, and wider health needs, supporting access to behavioral health care and other critical services. Coordinators help users understand their insurance coverage, find network providers, assist with scheduling appointments, and provide access to services.

### Soluna Implementation and Outreach

As part of Soluna’s rollout in California, implementation included free promotional materials provided to schools and community organizations to support awareness and youth engagement. These materials were informed by early usability testing and feedback on terminology used in outreach and engagement efforts, with attention to accessible, nonclinical language where appropriate.

Materials included posters and flyers displaying QR codes linking directly to the platform, wallet-sized cards that youth could discreetly take with them, stickers, and phone “pop-its” (small, silicone, tactile phone accessories that attach to the back of a smartphone). Soluna staff were actively involved in implementation efforts, including hosting informational presentations and trainings for staff, participating in school- and community-based events, and conducting in-person outreach activities, such as tabling at community events and school sites.

This study focused on stakeholders’ perceptions of Soluna’s implementation in their settings and their observations of how the platform impacted youth and their schools or organizations. Because Soluna accounts were designed for youth aged 13-25 years, staff did not have user access to the platform and were not expected to provide direct user experience data; however, demonstration versions were available to support training, outreach, and stakeholders’ understanding of the platform.

### Recruitment and Participants

Participants were recruited via convenience sampling. Recruitment procedures did not differ across stakeholder groups; all contacts were emailed using the same process. Surveys were distributed via email in February and April 2025 to key stakeholders whose contact information was already held by the Soluna implementation team through previous engagement and communication. The distribution list included representatives from K-12 schools, community-based organizations, higher education institutions, mental health care providers, and other youth-facing entities.

The initial survey invitation was sent to 5539 California-based contacts, with a 25.17% open rate and a 7.96% click-through rate to the survey link. A second email was sent to 1300 individuals who opened the initial invitation but did not click through to access the survey; this follow-up email had a 72.31% open rate and a 7.13% click-through rate. No additional emails were sent to individuals who did not open the initial invitation.

All recipients received the same survey invitation and study information. The survey link directed respondents to a single instrument that branched automatically based on whether they identified as school- or community-based stakeholders. A total of 91 individuals completed the survey; 14 were deemed ineligible based on the eligibility screener. Interview participation was open to all eligible survey respondents who volunteered. Interviews were scheduled on a first-come, first-served basis, and recruitment concluded once thematic saturation was reached.

### Data Collection

Before accessing the survey, participants completed screening questions to confirm eligibility: they had to be at least 18 years of age, reside in the United States, be able to read and write in English, and serve as frontline staff (directly interacting with youth) at a school- or community-based organization where the Soluna platform is offered. Eligible participants then completed the survey and indicated their willingness to provide further feedback via an interview. Those who expressed interest were invited to participate in one-on-one semistructured interviews conducted via a virtual video meeting (Zoom Communications). Interviews were conducted between February and April 2025 and were audio recorded and transcribed for analysis. Transcripts were deidentified and assigned unique study IDs before coding. These interviews lasted up to 30 minutes, and questions were tailored to the stakeholders’ context; for example, the interview protocol was slightly adapted for school-based stakeholders (focusing on youth within their schools) vs community-based stakeholders (focusing on youth engagement in their organization).

Interviews were conducted by the lead author, a female research scientist with a PhD and formal training in qualitative research. At the time of the study, she was working as a research scientist. No previous relationships existed between the interviewer and participants before study commencement, and no nonparticipants were present during interviews. Participants were informed that the interviewer was a research scientist conducting the study on behalf of Soluna to understand school- and community-stakeholder perspectives on implementation, adoption, and perceived impact of the platform. The interviewer approached the analysis reflexively. While professional interest in digital mental health research may have shaped analytic attention, no a priori assumptions about expected findings were imposed, and themes were grounded in participants’ accounts through iterative engagement with the data and memo writing. Field notes were taken during and immediately after each interview and reviewed following each interview to support analytic reflection.

### Measures

The cross-sectional survey was investigator-developed by the study team and informed by the CFIR and RE-AIM frameworks [18,19] to capture both implementation factors and stakeholder perceptions of the Soluna platform. Questions included participant demographics, stakeholder roles, perceptions of the platform’s impact on youth well-being and school or community environments, and the importance of various facilitators and barriers to adoption. Items were adapted for school- and community-based contexts to reflect stakeholders’ direct experiences. The survey was administered via Google Forms. Multimedia Appendix 1 provides the exact survey items and response options.

Semistructured interview questions (Textbox 1) were developed by the study team to expand on survey findings and to elicit in-depth perspectives on implementation experiences. The interview guide was informed by CFIR and RE-AIM domains and designed to explore perceived impact, adoption, facilitators and barriers, and implementation needs in real-world settings [18,19]. The guide was reviewed internally by researchers with qualitative and implementation science expertise and refined through informal pretesting and team-based review before data collection. Minor adaptations were made to tailor questions for school- vs community-based stakeholders, while maintaining consistency in core domains across interviews.

**Semistructured interview guide used with school- and community-based stakeholders.**

**Introduction:**

- What is your role at the school or organization, and how do you interact with students or youth?

**Facilitators:**

- What school-wide or community organization–wide factors have supported Soluna’s integration?
- Which Soluna features have been most effective for student or youth engagement and mental health?
- How has Soluna influenced engagement across different student or youth demographics?

**Barriers:**

- What challenges prevent students or youth from effectively using Soluna?
- Can you identify particular challenges faced by different student or youth demographics (eg, grade level, gender, ethnicity, or socioeconomic status) that hinder their engagement with Soluna?
- What school-wide or community organization–wide barriers make it harder to integrate Soluna?
- If you could improve one aspect of Soluna’s implementation, what would it be?

**Perceptions and feedback:**

- What are your overall thoughts on Soluna and its impact on school or community organization culture?
- What feedback have you heard from students or youth about Soluna?

**Personal experience and support:**

- Have you explored Soluna yourself?
- Would additional training help you support students or youth?
- How has Soluna affected your workload?

**Closing:**

- Any final thoughts on Soluna’s impact, challenges, or successes?

### Data Analysis

#### Survey Data

Survey responses were analyzed in SPSS (version 29; IBM Corp [28]). Descriptive statistics summarized stakeholder perceptions of the Soluna platform. Frequencies and percentages were computed for categorical variables, while means and SDs were calculated for continuous variables.

#### Qualitative Interview Data

Qualitative analyses were conducted in Dedoose (SocioCultural Research Consultants, LLC [29]). The lead author analyzed semistructured interview transcripts using inductive-deductive reflexive thematic analysis [30,31]. As coding was conducted by a single researcher, rigor was supported through reflective practices (eg, memo writing and iterative engagement with the data), grounding theme development in participant quotations, and discussing emerging themes with a coauthor. The interview questions (Textbox 1) guided the initial coding phase, while additional codes were developed based on themes that emerged from the interviews. Survey and interview data were analyzed separately and then triangulated during interpretation by examining how qualitative themes aligned with or contextualized survey findings. The lead author read each transcript in its entirety, applied descriptive labels to key excerpts, and then organized similar codes into broader categories that captured stakeholder perspectives on barriers, facilitators, and implementation of the Soluna platform. From these categories, high‑level findings were extracted that directly addressed the research aims and highlighted the key factors influencing the platform’s real‑world adoption.

### Ethical Considerations

This study was approved by the BRANY Institutional Review Board (protocol no 24-086-1708). All participants provided informed consent electronically. Before accessing any survey items, individuals were required to read an online consent form and select “I agree to participate” to proceed. Survey responses were anonymous, and identifying information was not collected within the survey. To receive compensation, participants were routed to a separate, unlinked form to provide their email address, ensuring that contact information could not be connected to survey data. Participants who volunteered for an interview had already received information in the consent form about the interview procedures, including that interviews would be audio recorded and that recordings would be deleted after transcription, with identifying details removed. Verbal consent was reconfirmed at the start of each interview. All data were stored on a secure, encrypted drive to protect confidentiality. Participants received a US $10 Amazon gift card for completing the survey and a US $25 gift card for participating in an interview.

## Results

### Participants

Seventy-seven stakeholders completed the survey: 54 school staff and 23 community staff. School respondents were primarily counselors or support personnel, while community respondents most often served as outreach workers or program directors. Overall, 83.1% of participants identified as women, and 50.6% identified as Hispanic or Latino(a). Stakeholder demographics are outlined in Table 1. Regarding implementation, Soluna had been offered at nearly half (49.4%) of schools and community organizations for 4-11 months. Following the survey, 17 stakeholders (12 school and 5 community) completed follow-up qualitative semistructured individual interviews.

**Table 1:** Demographic characteristics of school and community stakeholders participating in the Soluna study (N=77).

Demographic characteristic			Value, n (%)
**Current role**			
	**School stakeholders (n=54)**		
		Other school staff (eg, behavioral health therapist, support coordinator, mental health counselor, social worker, and wellness coach)	36 (66.70)
		Guidance counselor	12 (22.20)
		Teacher	3 (5.60)
		Principal	2 (3.70)
		School nurse	1 (1.90)
		Outreach staff	6 (26.10)
		Director or assistant director	5 (21.70)
	**Community stakeholders (n=23)**		
		Other (eg, program coordinator and social service worker)	4 (17.40)
		Counselor or therapist	2 (8.70)
		Manager or supervisor	2 (8.70)
		Peer specialist	2 (8.70)
		Case manager	1 (4.30)
		Administrative support staff	1 (4.30)
**Years working as school or community staff (years)**			
	0-2		11 (14.50)
	3-5		23 (30.30)
	6-9		15 (19.70)
	≥10		27 (35.50)
**Length of time Soluna has been offered at the school or community organization**			
	1-3 months		5 (6.50)
	4-6 months		16 (20.80)
	6-11 months		22 (28.60)
	1 year		17 (22)
	>1 year		17 (22.10)
**Gender**			
	Women		64 (83.10)
	Men		12 (15.60)
	Nonbinary or nonconforming		1 (1.30)
**Race or ethnicity**			
	Hispanic, Latino, or Latina(a)		39 (50.60)
	White		22 (28.60)
	Multiple races		10 (13)
	Black or African American		2 (2.60)
	Asian		1 (1.30)
	Native Hawaiian or Pacific Islander		1 (1.30)
	Native American or Alaska Native		0 (0)
Age (years), mean (SD; range)			37.45 (10.30; 22-60)

### Quantitative Survey Findings

#### Stakeholder Perceptions of Impact

Quantitative survey results offer insight into stakeholder perspectives on Soluna’s implementation and impact. Table 2 summarizes the proportion of respondents who agreed with each survey statement. Overall, survey responses reflected strong stakeholder endorsement of Soluna. Most stakeholders reported feeling comfortable referring youth to the platform (89.6%). The majority also agreed that the platform contributes positively to youth well-being (71.4%), addresses general mental health needs (76.6%), and is a safe and confidential resource (84.1%). While fewer stakeholders agreed that Soluna facilitates communication between youth and staff about mental health (62.2%) or supports a positive climate and culture (56.9%), these findings still indicate a favorable perception of the platform’s impact across schools and communities.

**Table 2:** Survey responses from school and community stakeholders on Soluna’s early implementation and impact (N=77).

Construct			Overall agreement (agree and strongly agree), n (%)
**Stakeholder perspectives of Soluna and its impact**			
	Positive well-being impact		55 (71.40)
	Referral comfort		69 (89.60)
	Positive climate and culture impact		44 (56.90)
	Positive behavior change		38 (49.30)
	Develop better emotional regulation skills		46 (59.70)
	Positive youth interactions with each other (community) and positive academic impact (school)		26 (33.50)
	Facilitate communication about mental well-being between youth and staff		48 (62.20)
	Effective addressing the mental health needs of youth		59 (76.60)
	Effective addressing the mental health needs of youth from racial or ethnic minority backgrounds		50 (64.90)
	Effective addressing the mental health needs of youth from gender minority backgrounds		49 (63.30)
	Confidence in the privacy and security of Soluna		59 (76)
	Effective in addressing or reducing bullying		22 (28.50)
	Contributed to the reduction in absenteeism		20 (26.60)
	Confidence Soluna safe platform to receive mental health support		65 (84.10)
**Top 3 features most valuable to youth^a^**			
	No cost		70 (91)
	Access during hours when traditional mental well-being supports are not available		35 (45)
	Easy to access		32 (42)
	No formal referrals needed		23 (30)
	No waiting list for professional support		18 (23)
	Reduced stigma around accessing anonymous support		14 (18)
	Safe place to receive mental health support		12 (16)
	Addresses a variety of mental health issues		5 (6)
**Facilitators of youth’s use of Soluna services**			
	Positive feedback from youth		57 (73.90)
	Strong support from school and community staff		44 (57.10)
	Engagement initiatives (workshops and information sessions)		37 (47.50)
	Accessible resources and training		57 (74)
	Ability to access mental health support through digital apps		67 (87)
	Confidential peer support		65 (84.10)
	Youth choice in what they engage in		66 (85.70)
	Youth need to understand what help they can get		65 (84.10)
**Barriers to youth’s use of Soluna services**			
	Lack of internet access		30 (39)
	Lack of time for use during school or in the community		34 (44.30)
	Stigma around mental health		50 (64.70)
	Lack of understanding of the platform		39 (50.80)
	Lack of awareness of the benefits of the platform		47 (61)
	Reluctance to engage with services		33 (42.30)
	Resistance from parents or guardians		28 (36.40)
	Privacy or confidentiality concerns		22 (28.60)
**Overall thoughts of Soluna**			
	On a scale from 1 to 5, how likely are you to recommend Soluna to other schools or organizations for students or youth? Answered “4” or “5”		73 (94.60)
	Have you directly referred any of the youth in your community to Soluna? (community) Answered “Yes”		65 (84.20)
	Have you directly referred any of your students to Soluna? (school) Answered “Yes”		65 (84.20)
	Overall, how pleased have you been with Soluna in terms of the goal to increase access to mental well-being support at your school or organization? Answered “Quite pleased” or “Very pleased”		67 (86.90)
	How confident are you that Soluna benefits school staff or organization staff in their role supporting students’ or youth mental well-being? Answered “Confident” or “Very confident”		64 (83.10)
	Do you think Soluna has helped to increase students’ engagement and concentration in school, and ultimately their academic outcomes? (school) Answered “Somewhat” or “Yes”		64 (88.70)
	Do you think Soluna has helped increase youths’ engagement in community-based programs? (community) Answered “Somewhat” or “Yes”		74 (95.70)
**Soluna implementation and integration**			
	Soluna presentation or event attendance: Answered “Yes”		57 (73.90)
**Reason for absence at Soluna presentation or event attendance**			
	Answered: “Not Aware”		15 (19.50)
	Answered: “Time Conflict”		3 (3.90)
	Answered: “Not Applicable”		25 (32.50)
Received an email from Soluna about the offering at the school or organization. Answered “Yes”			65 (84.40)
Read email: Answered “Yes”			63 (81.80)
Reason for not reading the email: Answered “Not Applicable”			43 (55.80)
School has signage (posters, fliers, etc) about Soluna: Answered “Yes”			60 (78)
Signage appropriately placed for maximum visibility: Answered “Yes”			50 (65)
**Soluna implementation and integration**			
	**The presentation or event was useful to me for understanding all that Soluna offers to students.**		
		Answered: “Agree”	54 (70.70)
		Answered: “Not Applicable”	13 (16.90)
	**The email about Soluna was useful for me in understanding all that Soluna offers to students.**		
		Answered: “Agree”	62 (80.20)
		Answered: “Not Applicable”	4 (5.20)
	**The Soluna posters and signage around the school are useful for students to learn about Soluna.**		
		Answered: “Agree”	55 (71.40)
		Answered: “Not Applicable”	13 (16.90)
	**I feel confident in knowing when to refer a student to Soluna.**		
		Answered: “Agree”	62 (80.40)
		Answered: “Not Applicable”	7 (9.10)
	**Having Soluna has contributed to a decrease in my workload.**		
		Answered: “Agree”	22 (28.60)
		Answered: “Not Applicable”	7 (9.10)

^a^The results are not mutually exclusive. Stakeholders selected up to 3 features they considered most valuable.

Each construct corresponds to a parallel survey item; for example, “Soluna has positively contributed to the overall well-being of our students” (school) and “Soluna has positively contributed to the overall well-being of youth in our community” (community).

#### Top Features

Across all stakeholders, the feature most frequently identified as most valuable for youth was Soluna’s free access (91%, n=70). This was followed by access during nontraditional hours (45%, n=35) and ease of access (42%). Other features stakeholders identified as valuable to youth included “no formal referral needed” (30%, n=23), “no waiting list for professional support” (23%, n=18), and “reduced stigma around accessing anonymous support” (18%, n=14). In addition, 16% (n=12) selected “a safe place to receive mental health support,” and 6% (n=5) selected “the platform treats a variety of issues” as a top feature. Because respondents could choose only up to 3 most valued features, percentages for some items are lower, and totals exceed 100%, but this does not necessarily indicate low perceived value for those features.

#### Facilitators and Barriers

Stakeholders identified several key facilitators of youth’s use of Soluna in their school or community (Table 2). The most widely endorsed was the ability to access mental health support through digital apps (87%, n=67). High agreement was also reported for the value of confidential peer support (84.1%, n=65) and providing youth with choice in what they engage in (85.7%, n=66). In addition, 74% of stakeholders agreed that accessible resources and staff training facilitated youth engagement with Soluna services. Stakeholder engagement initiatives, such as workshops and information sessions, were less frequently endorsed as facilitators (47.5%, n=37).

Stakeholders identified several common barriers to youth use of Soluna in their school or community. The most frequent barriers endorsed were stigma around mental health (64.7%, n=50) and lack of awareness of the platform’s benefits (61%, n=47). Nearly half (44.3%, n=34) reported that lack of time for youth’s use, either during school hours or at their community organization, was a barrier, and 39% (n=30) identified internet access issues as a limiting factor.

#### Overall Adoption and Satisfaction

Nearly all stakeholders reported they would recommend Soluna (94.6%, n=73) and had already referred to youth (84.2%, n=65). Overall satisfaction with Soluna was high (86.9%, n=67), and 83.1% (n=64) were confident that Soluna benefits staff in supporting student or youth mental well-being. The study also explored potential downstream effects on youth concentration and engagement. The majority of stakeholders perceived that Soluna had improved youth concentration in school (88.7%, n=64 indicated somewhat or yes) and engagement in community-based programs (95.7%, n=74 indicated somewhat or yes).

#### Implementation and Integration

Most stakeholders had attended a Soluna presentation or event (73.9%, n=57), received (84.4%, n=65), and read (81.8%, n=63) related emails, and reported visible poster signage in their schools or communities (78%, n=60). The majority found these communication channels useful for understanding Soluna (presentations: 70.7%, n=54 and emails: 80.2%, n=62) and felt confident in knowing when to refer youth to Soluna (80.4%, n=62). A more distal impact examined was staff workload, and 28.6% (n=22) of stakeholders reported that Soluna had already begun to reduce their workload during early implementation.

#### Qualitative Interview Findings

Qualitative analysis of 17 stakeholder interviews revealed three main categories with associated themes: (1) early impact, (2) facilitators to successful real-world implementation and adoption, and (3) barriers to successful real-world implementation and adoption. Themes are presented in order of frequency (n), with full counts and example quotes available in Multimedia Appendix 2.

### Early Impact

#### Platform Impact on Youth From Diverse Backgrounds

Stakeholders reported that Soluna had a positive impact on youth, noting that it was especially important for those from diverse contexts, including minoritized, rural, and low-income communities, where access to mental health resources was limited or conversations about mental health were often stigmatized.

We don't have resources out here for our students. So, having something literally at their fingertips has been very beneficial. It's a small community, and it's on the outskirts of the county. So apps, like Soluna, are just so important.

School Stakeholder #3, Female

#### Platform Impact on Stakeholder Workload

Stakeholders shared that they appreciated the platform as a valuable resource for supporting youth, particularly during times of staff shortages or high demand, even though it did not directly reduce their broader workload. Stakeholders appreciated that the platform allowed them to continue offering youth a form of support during times of high demand.

I think, for us it hasn't necessarily changed our workload, but it's an added service that we can't provide…with Soluna they have that option to still reach out to somebody. It's been an added benefit to what we're already doing.

School Stakeholder #2, Female

I feel like it's just helpful to offer another option. We have around 1,200 students and there's only four of us that get to speak with students. Our students' needs are very high. So it's nice to offer something else for the students.

School Stakeholder #10, Female

### Facilitators to Successful Real-World Implementation and Adoption

#### Materials and Merchandise From Soluna

Stakeholders noted that Soluna’s promotional materials, such as stickers, pop-its, and posters, helped raise awareness and pique the youth’s interest in the platform. These materials served as tangible reminders to explore the platform, with stickers and pop-its being especially effective in catching youth attention and sparking curiosity.

Things that I think connected them was when we had the stickers and little phone pop things that they had that they could put on their phone. Those kind of things. Kids like free swag. And they were like, ‘okay, free stuff. We're cool with that.’ But then, when they realized it was free stuff that had kind of a meaning behind it.

Community Stakeholder #11, Female

I was giving out stickers, and then a month later I was talking to a student about something, and she said, ‘Oh, yeah, I use that tool from the app you told me about.’ I'm like ‘Oh!’ And at that point we hadn't really dove deep into it. And she was like ‘You were giving out free stickers on the picture day’, and she had taken it upon herself, downloaded the app and was using the toolkit.

School Stakeholder #4, Female

Stakeholders highlighted the value of the promotional materials provided by Soluna, noting that having ready-to-use resources supported outreach efforts and made it easier to introduce the platform to youth. Materials such as posters, flyers, QR codes, and other take-home items were described as practical tools that could be easily distributed during events or during one-on-one interactions. Stakeholders also emphasized the importance of having materials available in Spanish, which supported outreach in communities with large Spanish-speaking populations.

They've given us all the material. So all you have to do is either print it out, and they'll give us flyers, so we just print them out in English and Spanish, and when we go do outreach with what they've given us, the youth coloring pages. So we take those to our events.

School Stakeholder #3, Female

It's been great with like the merch. I think it really helps just having something physical, tangible that I can give to students. You're like, “Hey, remember, Soluna, is this great app?”

School Stakeholder #10, Female

#### Platform-Specific Factors

Stakeholders highlighted several platform features that made it attractive to youth. The platform’s confidentiality was a major influence, as stakeholders expressed that youth valued the ability to access mental health resources anonymously.

I did have a learner say that they've used it a couple of times, so they really liked it. They like the privacy, being anonymous, not having any trail left back to them. [School Stakeholder #9, Male] Another stakeholder shared: They love the confidentiality.

School Stakeholder #2, Female

In addition, the platform’s digital format, offering 24/7 access, was crucial for youth who might not have access to mental health services outside of school hours.

The app and all of the features. They can use it, you know, at night time, when they need to.

School Stakeholder #17, Female

Stakeholders emphasized the platform’s free nature, noting that youth were often surprised by the range of features available at no cost.

It was easy to use and it was really just accessible. And she [student] was just really surprised of how much you guys were able to offer through a free app.

School Stakeholder #6, Male

Stakeholders emphasized that youth aged 13 years and older could access Soluna without parental permission, which they viewed as a significant advantage over other services that require signed parental release forms. They felt this reduced a barrier for youth who might otherwise hesitate to seek support due to concerns about parental resistance.

So I know for sure Hazel Health is telehealth, and it's therapy. And the students need to have a parent release form while Soluna doesn't. So that's a big advantage. For the students to access services without parent permission. Especially if they are 13 and older.

Community Stakeholder #2, Female

#### Referrals

Stakeholders frequently referred youth to the platform, particularly in situations where mental health resources were constrained, such as during school session breaks or in areas with high demand for mental health services. Referrals were particularly effective in getting youth to explore the platform during times when other support options were limited.

Even trying to find, therapy places, clinics, there are not a lot of options close for our community. So I have been telling the students that they can use the app to chat with a coach.

School Stakeholder #9, Female

Because of the difficulty and access to care, I end up with a lot of teenagers who didn't have a therapist who was a good fit, or didn’t really get the coping skills they were looking for. So once Soluna was offered, I do the same, vetting like I do with any other outside community resources.

Community Stakeholder#8, Female

#### Stakeholder Presentation of Soluna

Stakeholders described their role in presenting the platform to youth as a crucial factor in its adoption. They found that emphasizing Soluna’s practical benefits, such as coping skills or solutions to specific challenges, including bullying or academic stress, was more effective than using clinical mental health terminology.

Sometimes I refer to it [Soluna] as a coping skills app. As a distraction, tool. And that has been received better. Because when I do say ‘mental health app’, students are like, ‘well, my mental health is fine’ or like, ‘I don't want to work on my mental health.’ So when I reframe it, it definitely does help.

School Stakeholder #13, Female

When I say life skills training then there is a different kind of opening.

Community Stakeholder #2, Female

#### Soluna Engagement Team

The involvement of Soluna representatives in providing training, presentations, and support was considered a significant facilitator in promoting the platform. Stakeholders appreciated the direct engagement and expressed a desire for continued support from representatives to help with ongoing adoption efforts.

I think what really helped in terms of getting youth involved with Soluna, and how we were able to really incorporate was the help from the Soluna ambassador. She just was really helpful and passionate about getting our students involved in Soluna.

Community Stakeholder #6, Male

In a perfect world, it would be great to have an actual human that we know is our person. We know it's helpful, and we know that students have liked it in the past. So if there was funding, resources to be able to have a representative here.

School Stakeholder #13, Female

#### One-on-One Instruction

Another key factor in promoting adoption was one-on-one instruction. Stakeholders reported that walking the youth through the platform, explaining its features, and demonstrating its benefits helped the youth feel more comfortable downloading and using the platform.

They [youth] like me walking through it, cause I also know…just this population you have to put it in front of them for them to even recall that it's there. So I’m intentionally like, ‘we're gonna look at this one and this one. And I'm gonna show you like, this is a multi-purpose app.’

Community Stakeholder #7, Female

#### Hear From Other Youth

Several stakeholders pointed to the importance of peer recommendations in encouraging youth to use the platform. Stakeholders explained that youth who had used the platform and found it helpful often shared their experiences with peers, motivating others to explore the platform.

I know that they've [youth] been mentioning Soluna as a resource in their workshops that they run at lunchtime and activities. And so it's obviously beneficial. If students are the ones talking about the app and not just adults. So I think that is key… I think that's really powerful when other youth see youth sharing something that's engaging to them, and helpful.

School Stakeholder #6, Female

She [teacher] had two girls who were having issues like bullies and things like that, and one of the girls was like, ‘well, here, download this on your phone.’

Community Stakeholder #15, Female

### Barriers to Successful Real-World Implementation and Adoption

#### Lack of Instructions

Several stakeholders mentioned that the lack of detailed instructions on the platform’s features and resources was a significant barrier to adoption.

I can't use it. Like telling someone ‘these are the steps you're gonna do’ is very different than actually doing it.

School Stakeholder #2, Female

I think we're in an era where kids look up everything on TikTok and so like just having very basic videos on like this is how you access this. This is how you set up a meeting would be super helpful for them just to feel like extremely confident in what they're doing, what they're setting up. I think that it is a lot of the anxiety of starting something new and doing this. So I definitely think videos or like just tutorials would help them a lot.

School Stakeholder #1, Female

#### Stakeholder Desire for Local Usage Data

Stakeholders expressed strong interest in understanding how many youth are using Soluna and the reasons behind their engagement. They felt this information would help them better facilitate adoption.

The biggest thing would be a count of like people that have downloaded it or used it, or something. You know, in my position I push a lot of information out to them, but to us, knowing that they are using it or seeing like if there's an influx going on would be helpful.

School Stakeholder #10, Male

#### Phone Restrictions (Parental and School Policies)

Stakeholders frequently cited restrictions on phone use both at home and at school as significant barriers to the platform’s use. Parental limits on screen time, along with youth being grounded or having limited phone access, often prevented them from using the platform when needed.

Now we're like a completely cell phone-free school. And so the students don't have access to put those things on their phone at school.

School Stakeholder #7, Female

I do know that certain students, especially when they get in trouble they get their phone taken away. But there's this underlying mental health issue. But now they can't access it.

School Stakeholder #9, Female

#### Lack of Awareness

A common barrier identified by stakeholders was a general lack of awareness among youth about the platform’s existence and its potential benefits. Stakeholders noted that youth are often inundated with numerous other apps and digital tools. Stakeholders also noted that some youth were skeptical about the platform being truly free, as they are typically accustomed to encountering hidden costs in digital apps.

It doesn't get recognized enough where on the app you're able to talk with a professional. It just seems relatively a little bit confusing to me, cause I was like ‘is it free’? It is a free option. I feel like that needs to be expressed a little bit more, because I feel like that's one of the biggest selling points of the Soluna app.

Community Stakeholder #6, Male

I just feel like everybody's kind of inundated with apps and there’s an oversaturation, I guess, in the market of similar products. So I think a lot of students and families, especially think that there's some sort of catch, you know.

School Stakeholder #7, Female

#### Mental Health Stigma

Stakeholders reported that mental health stigma was a barrier to youth engagement. This included stigma at the community and cultural level, as well as the youths’ own discomfort with seeking help or participating in practices such as counseling, meditation, or breathing exercises. Stakeholders shared that this stigma often deterred some youth from seeking help or using mental health tools, including the platform.

I think the main barrier is the stigma of needing someone to talk to, or needing to speak to a counselor or that kind of stuff. There's so many youth we have, particularly in lower-income communities where it's still a stigma and seen as a weakness…You know, because in the Hispanic community things like depression and anxiety, we don't talk about those things. That's just not something that we do.

Community Stakeholder #12, Female

## Discussion

### Overview of Findings

The purpose of this mixed methods study was to (1) examine stakeholders’ views of the platform’s perceived impact on youth mental health and well-being and on school or organizational contexts, (2) identify barriers and facilitators influencing early implementation and adoption in real-world school and community settings, and (3) explore downstream or distal effects of platform implementation, including perceived changes in staff workload and youth engagement in school and community settings. Both quantitative and qualitative data revealed important barriers and facilitators to the platform’s implementation and adoption in the first year of the platform’s launch in California, along with valuable suggestions for implementation strategies for similar digital well-being platforms. These insights contribute to a deeper understanding of how digital mental health platforms can be more effectively integrated into youth support systems, ensuring broader engagement and sustained use. Many stakeholders perceived the platform as positively impacting youth well-being and supporting youth mental health. These findings can be used to inform future research and guide practical strategies for the sustainable integration of digital mental health tools into everyday settings for youth, ultimately enhancing overall youth wellness and support.

### Perceived Benefits for Youth Mental Health and Well-Being

Both the quantitative survey and qualitative interviews highlighted that stakeholders felt the platform was a valuable tool for addressing youth mental health needs. The survey responses suggested that the platform contributed positively to youth well-being, while the qualitative interviews further elaborated on how the platform provided a critical resource, especially for youth in underserved or low-resource settings. Stakeholders perceived the Soluna platform as having a positive impact on youth mental health.

### Impact

While the stakeholder survey assessed specific perceived impacts on youth well-being, mental health, and staff workload, the qualitative interviews relied on a single open-ended question, capturing where stakeholders perceived Soluna had an impact but not necessarily the full range of possible impacts. Stakeholders felt that Soluna had a significant impact on youth from diverse backgrounds, particularly in underserved areas, where stakeholders said it filled a crucial gap in mental health services. For underresourced communities, digital mental health platforms can be crucial in bridging gaps in mental health services, offering accessible, confidential, and free support where traditional resources are lacking [32]. To maximize their impact, digital mental health companies should focus on partnerships with local organizations and explore funding opportunities to ensure these platforms reach the communities that need them most while maintaining financial sustainability. Soluna’s approach of working closely with schools and community organizations during early implementation shows that such collaboration is critical for driving adoption and increasing reach.

Our findings suggest that digital mental health platforms can play a critical role in supporting school and organizational staff by providing an accessible way to extend mental health support to youth, especially in environments where resources are scarce. Although stakeholder workload was not a primary outcome, it was examined as an exploratory indicator of potential distal effects of platform implementation. While roughly one-third of stakeholders reported a perceived reduction in workload in the quantitative survey, qualitative findings suggested early ripple effects of implementation in school and community settings.

### Facilitators to Successful Real-World Implementation and Adoption

Stakeholders identified promotional materials as critical to Soluna’s successful implementation. Free resources such as posters, QR codes, and cards raised awareness in classrooms and offices, while cards provided a discreet way for youth to access information without directly asking for help. Spanish-language versions further expanded reach in diverse communities. Digital mental health platforms seeking broader adoption should consider a similar approach, offering free promotional materials to schools and community organizations. Branded items such as stickers and pop-its were also effective in sparking curiosity and increasing awareness of the platform. Studies suggest that extrinsic incentives can help spark initial interest in mental health tools, especially when the tools are seen as personally relevant and useful [33]. For digital mental health companies, stakeholders identified that branded items may offer an approachable entry point that helps normalize mental health conversations and raise awareness, making them a worthwhile strategy to consider for encouraging early engagement.

Platform-specific features also supported adoption. Stakeholders emphasized confidentiality, anonymity, 24/7 access, no cost, and no requirement for caregiver permission as central factors influencing youth engagement. These findings are consistent with evidence that cost and parental consent remain major barriers to care [33,34]. Ensuring platforms remain free and self-accessible is therefore essential to reaching youth who may otherwise go without support.

Direct referrals further strengthened adoption, particularly during times when traditional services were less available, such as school breaks or in high-demand settings. Youth were also likely to recommend the platform to peers, creating additional pathways for engagement. This aligns with evidence that peer influence strongly motivates youth to engage with mental health resources [35], and research suggests direct referrals are associated with lower dropout from digital mental health tools [36]. Ohio’s youth peer advocate initiative trains young people with lived experience to share resources and guide peers toward mental health support, helping to build awareness and trust [37]. Adapting similar peer referral models for digital platforms may offer a promising strategy to boost youth engagement and sustained use. Soluna is applying a similar approach through its Youth Ambassador Program and upcoming peer-to-peer content-sharing features. Future research should evaluate the impact of these initiatives on stakeholder perceptions, as well as on real-world engagement and retention with the platform.

Stakeholders found that using language that emphasized the practical benefits of the platform, such as coping skills and solutions to specific challenges, such as academic stress or bullying, resonated more effectively with youth than framing the platform purely as a mental health tool. Research suggests that stigma often discourages youth from seeking mental health support, and that they prefer tools offering immediate, tangible benefits such as symptom relief, mood improvement, and self-reflection [10,38]. Using nonclinical language and emphasizing real-world relevance may help digital mental health platforms feel more approachable and meaningful in young people’s daily lives [12].

Personalized support to schools and community organizations at multiple levels was key to successful implementation and engagement with the Soluna platform. Training, presentations, and continued engagement from Soluna staff equipped school and community partners to introduce the platform effectively. One-on-one instruction during initial use was especially influential in helping youth feel comfortable returning to the platform. These findings reinforce the importance of ongoing professional support to sustain the integration of digital mental health tools [39]. Digital mental health companies should consider investing in ongoing, personalized support through trained individuals, either in person or through digital channels, who can provide instruction, presentations, and direct engagement with schools and community organizations. Experience from Soluna in California suggests that combining low-barrier access to digital platforms with sustained, meaningful in-person engagement and localized partnerships is central to achieving reach, adoption, and maintenance [19].

### Barriers to Successful Real-World Implementation

Several barriers to the real-world implementation of the Soluna platform were identified. Stakeholders expressed a need for clear instructions surrounding the platform features and resources, along with a demo mode to allow both youth and staff to explore the platform before committing to use. Stakeholders expressed that without a way to fully experience the platform’s features, it was difficult for them to promote it effectively to youth. Ensuring sufficient onboarding support is essential to enable broader user engagement and facilitate the wider implementation of digital mental health interventions. For instance, a systematic review identified that technical issues are common barriers to user engagement with digital mental health interventions [40]. In addition, program usability, including clear instructions and accessible design, significantly influences young people’s engagement with web-based mental health interventions [10]. Incorporating comprehensive onboarding processes, such as interactive demos and clear instructional materials, could facilitate the adoption and sustained use of digital mental health platforms among youth [41]. Learning from early implementation and how users engage with the digital tools can also provide a feedback loop to adapt and improve engagement materials and onboarding flows.

Several stakeholders desired metrics on how many youth were using the platform at their schools or organizations. Without this data, it was difficult for stakeholders to assess whether their efforts were successful or to determine how effectively the platform was being used. This lack of insight also made it challenging for stakeholders to identify patterns or adjust their strategies to improve engagement. However, data sharing is complicated by the platform’s confidential nature, as users are not linked to a specific school, and there are limitations on sharing ZIP code–level data due to privacy concerns. As a potential solution, digital mental health companies could implement QR code tracking or in-app self-report features to help identify referral sources while preserving user anonymity. Companies can also share aggregate, non–school-specific reports with schools and organizations that include actionable recommendations based on overall data trends to inform outreach and implementation strategies. Such sharing should be coordinated with the contract payer to determine what engagement data can be appropriately shared.

There were also logistical barriers identified by stakeholders, such as phone restrictions imposed by schools, which further hindered platform engagement. These findings underscore the need to address external constraints, such as school policies, limited access to devices, and the importance of educating parents about the platform when implementing digital mental health platforms for youth. Meta-analytic evidence shows that school-based mental health interventions are most effective when delivered frequently and integrated into the school day, highlighting the value of allowing structured access to digital platforms during designated periods [42]. Schools should consider integrating structured access to digital mental health platforms during designated periods. In addition, schools could offer virtual or social media–based parent education to increase awareness of how digital mental health tools support youth and encourage access during times of distress. Such efforts can build shared understanding and reduce barriers to help-seeking [43].

Low awareness among youth was another barrier during the first year of launch. Some youth were unaware of Soluna’s availability or benefits, which limited early adoption. As with many digital platforms, an initial adoption period is expected, during which awareness and trust build over time [44]. One option to help promote awareness is to involve trusted adults, such as school or library staff, in sharing information about the platform. For example, public libraries often conduct weekly school outreach, youth-focused events, and partnerships with schools, park districts, and mental health clinics to promote programs and connect youth to resources [45]. These strategies can help make digital tools more visible and accessible to youth in familiar, supportive environments.

Finally, stigma remained a significant barrier, especially in communities where mental health is not openly discussed. Stakeholders from certain cultural communities noted that stigma discouraged youth from seeking help. This is consistent with research showing that stigma disproportionately affects youth from racial and ethnic minority backgrounds [46]. Schools and community organizations should collaborate with digital platforms providing well-being or mental health services on education-based, culturally tailored interventions led by trained facilitators to reduce stigma and normalize mental health discussions among youth [47].

### Recommendations for Mental Health Companies and Industry Professionals

Based on the findings from this study, we make several recommendations for digital mental health companies developing platforms for youth and for researchers involved in their evaluation. These recommendations aim to bridge the gap between research insights and real-world implementation challenges, ultimately improving the design and effectiveness of digital mental health tools for youth. Table 3 outlines key issues identified in the study and provides tailored recommendations for both digital mental health companies and industry professionals (eg, behavioral scientists and product engineers).

**Table 3:** Recommendations for digital mental health companies and industry professionals informed by stakeholder interviews.

Issue or topic	Recommendation for industry professionals	Recommendation for digital mental health companies
Stigma and mental health framing	Collaborate with product teams to develop and test messaging that focuses on coping skills and well-being to reduce stigma.	Frame the platform in terms of skills building (eg, coping strategies and emotional regulation) and mental well-being rather than focusing on clinical mental health language; develop culturally tailored campaigns that normalize mental health support.
Low awareness among youth	Partner with communications and IT teams to integrate platform promotions into student portals, learning systems, and routine announcements.	Invest in youth-led, peer-to-peer, and influencer-driven campaigns; build social media toolkits co-designed with youth to increase reach and relatability.
Lack of usage data for stakeholders	Use QR codes and in-app self-report prompts to track referral sources while preserving anonymity.	Share aggregate, non–school-specific reports with schools and organizations that include actionable recommendations based on overall data trends to guide outreach and implementation.
Importance of direct support and training	Coordinate with program managers to establish a cadence of representative-led orientations and refreshers throughout the year.	Fund and train a team of community engagement specialists to deliver school-based and community training sessions (live or virtual) and build local relationships.
Promotional materials and visibility	Coordinate with outreach and development teams to distribute merchandise (eg, stickers and QR codes) during events or in high-traffic areas.	Create bundled outreach kits with tiered options (free for low-resource settings and discounted for others); include multilingual and culturally relevant assets.
Effective messaging to youth	Test and deploy notifications and content that emphasize solving everyday challenges (eg, bullying and academic stress) instead of mental health–focused language.	Conduct A/B testing on notifications and content that center on daily life skills and reframes help-seeking as a strength and self-improvement.
Referral as a key facilitator to adoption	Support and equip peers to share digital mental health tools, and encourage trusted adults (eg, educators, counselors, and youth program staff) to actively promote them.	Streamline referral pathways through digital tools, QR codes, and cobranded materials; consider referral incentive programs.
Logistical barriers (eg, phone restrictions)	Collaborate with product engineers to explore ways to expand access to the platform through shared devices (eg, Chromebooks) in schools, libraries, or community centers.	Explore partnerships with schools to ensure that the platform is accessible through shared devices in classrooms or school libraries.
Need for demo mode and onboarding support.	Work with product engineers to create real-time support systems (eg, live chat and interactive tutorials) that cater to individual needs.	Develop an interactive demo version or sandbox mode to showcase key features.

### Limitations

This study has limitations that should be considered. First, the findings are based on stakeholder reports, and youth perspectives were not directly included in the data collection. This may influence interpretation by misrepresenting youths’ actual experiences of the platform, particularly in ways that are not readily observable by adult staff. While outside the scope of this study, future research should complement stakeholder perspectives by directly assessing youth experiences and examining changes in youth mental health outcomes to more fully understand the platform’s effectiveness. In addition, the study relied on self-reported data from stakeholders, which could introduce bias, such as social desirability or recall bias. Another limitation is the self-selection bias in participant recruitment, which could influence interpretation by making implementation appear more feasible or less challenging than it may be in settings facing greater barriers. Furthermore, the surveys and interviews were conducted between February and April 2025. Engagement initiatives related to the platform evolved after this period, although overarching implementation strategies remained similar. These changes in engagement efforts and promotional strategies over the first year of implementation may not be reflected in the results, and future research should consider these updates when assessing the platform’s impact. In addition, interview data were coded by a single researcher, which did not allow for intercoder comparison, though qualitative findings were interpreted alongside survey results to support analytic rigor. The study focused on a specific platform deployed in California, limiting the generalizability of the results to other regions or platforms. Future research should aim to include a broader range of stakeholders, incorporate youth feedback, and consider longitudinal studies to evaluate the long-term effectiveness of digital mental health platforms.

### Conclusions

This study provides valuable insights into how key stakeholders from schools and community organizations perceive the impact, implementation, and adoption of a free, confidential digital mental health platform for youth in California during its first year of implementation. These insights contribute to the implementation of digital mental health tools and can help guide future efforts to better meet the perceived needs of both youth and the organizations that serve them, particularly in underresourced settings. In addition, the findings inform product development, marketing, and customer support teams within digital mental health companies, offering suggestions for practical ways to improve platform features, outreach strategies, and support systems to facilitate broader adoption and successful implementation, learning from a real-world example of a scaling digital mental health platform.

## Appendix

Multimedia Appendix 1 School and community stakeholder quantitative survey items.

Multimedia Appendix 2 Themes and example quotes.

## Abbreviations

CFIR: Consolidated Framework for Implementation Research
RE-AIM: Reach, Effectiveness, Adoption, Implementation, and Maintenance

## Data Availability

The datasets generated and analyzed during this study are not publicly available due to proprietary restrictions and confidentiality agreements. However, aggregated and anonymized data are available from the corresponding author upon reasonable request.
