# Supplementary material for: School and Community Stakeholder Perceptions of a Free, Confidential Digital Mental Health Platform (Soluna): Mixed Methods Study Examining Barriers and Facilitators to Real-World Implementation at Scale and Early Impact
Source: JMIR Form Res. 2026 Mar 4;10:e82864. doi: 10.2196/82864 (PMC13000374; doi:10.2196/82864)
Supplement: Multimedia Appendix 1 [file formative_v10i1e82864_app1.docx]

## **School-Based Stakeholder Survey**

Unless otherwise noted, agreement items used a 5-point Likert scale
(Strongly disagree, Disagree, Neutral, Agree, Strongly agree).

###

### **Perceived Impact of Soluna on Students and School Environment**

*Please indicate the extent to which you agree or disagree with the following statements:*

- Soluna has positively contributed to the overall well-being of our students.
  I feel comfortable directing my students to Soluna as a resource for their mental well-being.
- Soluna has had a positive impact on the overall school climate and culture.
- I have observed positive changes in students’ behavior since the implementation of Soluna.
- Soluna has helped students develop better emotional regulation skills.
- Soluna has positively influenced students’ grades and completion of assignments.
- Soluna has helped facilitate students and teachers to openly communicate about mental well-being.
- Soluna has been effective in addressing the mental health needs of our students.
- Soluna has been effective in addressing the mental health needs of students from racial/ethnic minority backgrounds.
- Soluna has been effective in addressing the mental health needs of students from gender minority backgrounds.
- I am confident in the privacy and security measures implemented by Soluna.
- Soluna is effective in addressing and reducing instances of bullying in my school.
- Soluna has contributed to a reduction in absenteeism among my students.
- I am confident that Soluna is a safe platform for students to receive mental health support.

### **Perceived Value of Soluna Features**

- *What are the top 3 features of Soluna that you think are most valuable to your students?*
  - No cost
  - Easy access
  - Access during hours where traditional mental wellbeing supports are not available
  - Reduced stigma around accessing anonymous support
  - Safe place to receive mental health support
  - Addresses a variety of mental health issues
  - No formal referrals needed
  - No waiting list for professional support
  - Other (please specify)

### **Facilitators of Youth Use of Soluna in Schools**

*Please indicate the extent to which you agree or disagree that the following factors facilitate youth use of Soluna services in your school:*

- Positive feedback from youth
- Strong support from staff
- Engagement initiatives (workshops, information sessions)
- Accessible resources and training
- Ability to access mental health support through digital apps
- Ability for youth to engage in confidential peer support
- Ability for youth to have a choice in what they engage in
- Youth have a place to understand what help they can get for their mental and behavioral health
- Other (please specify)

### **Barriers to Youth Use of Soluna in Schools**

*Please indicate the extent to which you agree or disagree that the following factors are barriers to youth use of Soluna services in your school:*

- Lack of internet access
- Lack of time for use during school hours
- Stigma around mental health
- Lack of understanding of the platform
- Lack of awareness of the benefits of the platform
- Reluctance to engage with services
- Resistance from parents or guardians
- Privacy or confidentiality concerns
- Other (please specify)

### **Implementation, Outreach, and Satisfaction**

- *On a scale from 1 to 5, how likely are you to recommend Soluna to other schools?*
  - Response options: 1 (Not likely at all) to 5 (Very likely)
- *Overall, how pleased have you been with Soluna in terms of increasing access to mental wellbeing support at your school?*Response options:
  - Not pleased at all
  - Not very pleased
  - Neither pleased nor displeased
  - Quite pleased
  - Very pleased
- Follow-up (open-ended): If pleased, why? If not pleased, why not?
- *How confident are you that Soluna benefits school staff in supporting students’ mental wellbeing?*Response options:
  - Not confident at all
  - Not very confident
  - Neither confident nor not confident
  - Confident
  - Very confident
- *Do you think Soluna has helped increase students’ engagement and concentration in school?*Response options:
  - - Yes
    - Somewhat
    - No
  - Follow-up (open-ended): Please explain
- *Did you attend a Soluna presentation or event?*
  - Response options:
    - Yes
    - No
  - If no:
    - I was not aware of the presentation/event
    - I had a conflicting event at the time of the presentation/event
    - I did not think it was relevant to me
    - N/A
- *Did you receive an email about Soluna at your school?*
  - Response options:
    - Yes
    - No
- *Did you read the email about Soluna at your school?*
  - Response options:
    - Yes
    - No
    - N/A
  - If no:
    - The email was too long
    - I did not think it was relevant to me
    - N/A
- *Does your school have Soluna signage (e.g., posters, flyers)?*
  - Response options:
    - Yes
    - No
- *Is the signage appropriately placed for maximum visibility?*
  - Response options:
    - Yes
    - No
    - N/A
- *I feel confident in knowing when to refer a student to Soluna.*
  - (Strongly disagree, Disagree, Neutral, Agree, Strongly agree)
- *Having Soluna at my school has contributed to a decrease in my workload.*
  - (Strongly disagree, Disagree, Neutral, Agree, Strongly agree)
- *Have you directly referred any students to Soluna?*
  - Yes
  - No

### **Demographic and Professional Background (School-Based Stakeholders)**

- *How many years have you been working as a school staff member (not just at your current school but at any school)?*
  - Response options:
    - Less than 1 year
    - 1 year
    - 2 years
    - 3 years
    - 4 years
    - 5 years
    - 6 years
    - 7 years
    - 8 years
    - 9 years
    - 10 years or more
- *What is your current role?*
  - Response options:
    - Teacher
    - Guidance counselor
    - Principal
    - Vice principal
    - School nurse
    - Superintendent
    - Other school staff
  - *If other school staff, please specify:* (open-ended)
- *How long has Soluna been offered to students at your school?*Response options:
  - - Less than 1 month
    - 1–3 months
    - 4–6 months
    - 6–11 months
    - 1 year
    - More than 1 year
- *Which gender identity best describes you? (Please select all that apply)*
  - Response options:
    - Woman
    - Man
    - Intersex
    - Non-binary / Non-conforming
    - Trans
    - Two-spirit
    - I prefer to self-identify
  - *If you prefer to self-identify, please specify:* (open-ended)
- *Which race/ethnicity best describes you? (Please select all that apply)*
  - Response options:
    - Hispanic or Latino/a
    - Asian or Asian American
    - Black or African American
    - Middle Eastern or North African
    - Native American or Alaska Native
    - Native Hawaiian or Pacific Islander
    - White or European
    - Another
  - *If another, please specify:* (open-ended)
- *Please select your age.*
  - Response options: 18 through 80+
- *Would you like to participate in a follow-up interview (up to 30 minutes) to tell us more about your thoughts on Soluna?*

## **Community-Based Stakeholder Survey**

Unless otherwise noted, agreement items used a 5-point Likert scale
(Strongly disagree, Disagree, Neutral, Agree, Strongly agree).

###

### **Perceived Impact of Soluna on Youth and Community Settings**

*Please indicate the extent to which you agree or disagree with the following statements.*

- Soluna has positively contributed to the overall well-being of youth in our community.
- I feel comfortable directing youth in my community to Soluna as a resource for their mental well-being.
- Soluna has had a positive impact on the overall culture in our community.
- I have observed positive changes in youth’s behavior since the implementation of Soluna.
- Soluna has helped youth in my community develop better emotional regulation skills.
- Soluna has positively influenced youth’s interactions with each other.
- Soluna has helped facilitate youth and community-based organization staff to openly communicate about mental well-being.
- Soluna has been effective in addressing the mental health needs of youth in our community.
- Soluna has been effective in addressing the mental health needs of youth from racial/ethnic minority backgrounds.
- Soluna has been effective in addressing the mental health needs of youth from gender minority backgrounds.
- I am confident in the privacy and security measures implemented by Soluna.
- Soluna is effective in addressing and reducing instances of bullying in our community.
- Soluna has contributed to a reduction in youth absenteeism in our community programs.
- I am confident that Soluna is a safe platform for youth to receive mental health support.

### **Perceived Value of Soluna Features**

- *What are the top 3 features of Soluna that you think are most valuable to youth?*
  - No cost
  - Easy access
  - Access during hours where traditional mental wellbeing supports are not available
  - Reduced stigma around accessing anonymous support
  - Safe place to receive mental health support
  - Addresses a variety of mental health issues
  - No formal referrals needed
  - No waiting list for professional support
  - Other (please specify)

###

### **Facilitators of Youth Use of Soluna in Community-Based Organizations**

*Please indicate the extent to which you agree or disagree that the following factors facilitate youth use of Soluna services in your organization.*

- Positive feedback from youth
- Strong support from staff
- Engagement initiatives (workshops, information sessions)
- Accessible resources and training
- Ability to access mental health support through digital apps
- Ability for youth to engage in confidential peer support (e.g., able to talk to others without being identified)
- Ability for youth to have a choice in what they engage in
- Youth have a place to understand what help they can get for their mental and behavioral health
- Other (please specify)

### **Barriers to Youth Use of Soluna in Community-Based Organizations**

*Please indicate the extent to which you agree or disagree that the following factors are barriers to youth use of Soluna services in your organization.*

- Lack of internet access
- Lack of time for use
- Stigma around mental health
- Lack of understanding of the platform
- Lack of awareness of the benefits of the platform
- Reluctance to engage with services
- Resistance from parents or guardians
- Privacy or confidentiality concerns
- Other (please specify)

### **Implementation, Outreach, and Satisfaction**

- *On a scale from 1 to 5, how likely are you to recommend Soluna to other community-based organizations for youth?*
  - Response options: 1 (Not likely at all) to 5 (Very likely)
- *Overall, how pleased have you been with Soluna in terms of the goal to increase access to mental wellbeing support at your community based organizations?*Response options:
  - Not pleased at all
  - Not very pleased
  - Neither pleased nor displeased
  - Quite pleased
  - Very pleased
- Follow-up (open-ended): If pleased, why? If not pleased, why not?
- *How confident are you that Soluna benefits staff in their role supporting youth mental wellbeing?*Response options:
  - Not confident at all
  - Not very confident
  - Neither confident nor not confident
  - Confident
  - Very confident
- *Do you think Soluna has helped to increase youths’ engagement in community-based programs?*Response options:
  - Yes
  - Somewhat
  - No
- Follow-up (open-ended): Please explain
- *Did you attend a Soluna presentation or event?*
  - Response options:
    - Yes
    - No
  - If no:
    - I was not aware of the presentation/event
    - I had a conflicting event at the time of the presentation/event
    - I did not think it was relevant to me
    - N/A
- *Did you receive an email about Soluna at your organization?*
  - Response options:
    - Yes
    - No
- *Did you read the email about Soluna at your organization?*
  - Response options:
    - Yes
    - No
    - N/A
  - If no:
    - The email was too long
    - I did not think it was relevant to me
    - N/A
- *Does your organization have Soluna signage (e.g., posters, flyers)?*
  - Response options:
    - Yes
    - No
- *Is the signage appropriately placed for maximum visibility?*
  - Response options:
    - Yes
    - No
    - N/A
- *I feel confident in knowing when to refer a youth to Soluna.*
  - (Strongly disagree, Disagree, Neutral, Agree, Strongly agree)
- *Having Soluna at my organization has contributed to a decrease in my workload.*
  - (Strongly disagree, Disagree, Neutral, Agree, Strongly agree)
- *Have you directly referred any of the youth in your community to Soluna?*
  - Yes
  - No

### **Demographic and Professional Background (Community-Based Stakeholders)**

- *What is your current role?*
- Response options:
  - - Teacher/Instructor
    - Administrative or support staff (e.g., account manager, administrative assistant)
    - Director/Assistant Director
    - Case Manager
    - Counselor/Therapist
    - Manager/Supervisor
    - Executive Officer
    - Outreach Staff
    - Peer Specialist
    - Volunteer
    - Other
  - *If other, please specify:* (open-ended)
- *How many years have you been working as a community-based organization (CBO) staff member (not just at your current organization but at any CBO)?*
  - Response options:
    - Less than 1 year
    - 1 year
    - 2 years
    - 3 years
    - 4 years
    - 5 years
    - 6 years
    - 7 years
    - 8 years
    - 9 years
    - 10 years or more
- *How long has Soluna been offered to youth at your community-based organization?*
  - Response options:
    - Less than 1 month
    - 1–3 months
    - 4–6 months
    - 6–11 months
    - 1 year
    - More than 1 year
- *Which gender identity best describes you? (Please select all that apply)*
  - Response options:
    - Woman
    - Man
    - Intersex
    - Non-binary / Non-conforming
    - Trans
    - Two-spirit
    - I prefer to self-identify
  - *If you prefer to self-identify, please specify:* (open-ended)
- *Which race/ethnicity best describes you? (Please select all that apply)*
  - Response options:
    - Hispanic or Latino/a
    - Asian or Asian American
    - Black or African American
    - Middle Eastern or North African
    - Native American or Alaska Native
    - Native Hawaiian or Pacific Islander
    - White or European
    - Another
  - *If another, please specify:* (open-ended)
- *Please select your age.*
  - Response options: 18 through 80+
- *Would you like to participate in a follow-up interview (up to 30 minutes) to tell us more about your thoughts on Soluna?*
