# Supplementary material for: School and Community Stakeholder Perceptions of a Free, Confidential Digital Mental Health Platform (Soluna): Mixed Methods Study Examining Barriers and Facilitators to Real-World Implementation at Scale and Early Impact
Source: JMIR Form Res. 2026 Mar 4;10:e82864. doi: 10.2196/82864 (PMC13000374; doi:10.2196/82864)
Supplement: Multimedia Appendix 2 [file formative_v10i1e82864_app2.docx]

| Categories | Themes | Example quotes |
| --- | --- | --- |
| Early Impact | Platform Impact on Youth from Diverse Backgrounds (n=11) | Our district and all of our schools are very rural. And so we really don't have any, literally, zero mental health. No brick and mortar or anything in our town. Outside of the counseling offices at our school sites, there's nothing for youth. So I think bringing that to the fingertips of people is a really big deal here in our community. [School Stakeholder #6, Female]  When you deal with communities like the Hispanic community, and sometimes even the African American community, mental health is not something that's discussed or talked about hardly at all. And then, if it is, it has a very negative connotation to it. So, like kids having that option to where they can just go to their phone, it made it more of a universal thing, less of a ‘You know we can't talk about it, but we can go here, and we can talk about it.’ It just gave them that option. So I think that is kind of what made it to where any kid can access it. [Community Stakeholder #12, Female] |
|  | Platform Impact on Stakeholder Workload (n=6) | I don't think it's impacted my workload at all, because I'm still like very involved, you know, with the students…it's just like another resource that I can refer them to… and like before the holidays I sent out a newsletter like, ‘Hey, we're gonna have a couple of weeks off, and here's one of the resources that you can use.’ So it provides peace of mind for me, versus like doesn't impact my workload. But peace of mind is wonderful. [School Stakeholder #4, Female]  Well…I can tell you that it's a game-changer for a lot of our counselors, because they can offer that immediately without waiting lists. So that is really something that is appreciated very much, you know. [Community Stakeholder #1, Female] |
| Facilitators to Successful Real-World Implementation and Adoption | Materials and Merchandise from Soluna (n=13) | The merch is huge like. It's just such a draw, and then it gets you having that conversation about it, whereas, like, if you're just bringing it up, and there's no visual with it, it just tends to be blah. [School Stakeholder #4, Female]  I want to highlight the promotional materials… when we asked for them, we got them pretty quickly...And the Spanish versions, too were really helpful, because we have a lot of Spanish-speaking students as well. The fast response and follow-through was really helpful in getting out the information quickly. [School Stakeholder #16, Female]  I love the fact that they offer free materials. That is a huge thing, because getting printed materials that are good quality, and not having to pay for it…materials and budget can be a barrier for a lot of schools. So the fact that the app is free if the materials are free. I think that would be like my biggest thing, I think that helps us. [School Stakeholder #9, Female] |
|  |  |  |
|  | Platform-Specific Factors (n=10) | I've seen that they like the fact that they can get someone quick. That they can do it in their own time, so they don't feel the stigma of having to go or get help. [School Stakeholder #15, Female]  They don't have to sign anything. It's confidential. They really appreciate that. [School Stakeholder #12, Female]  The coaching. So some so that has worked for students just because they're either in the phase of like, I don't want therapy, but I'm willing to try like chatting to someone, even if it's not in a therapeutic setting, just to see how it is, because it's like no strings attached, and they could at any point like stop it. [School Stakeholder #13, Female]  And the gamified approach to it. I've heard good responses that students are engaging in it, and that they like it. [School Stakeholder #6, Female]  They love that it's an app. It's easy to use. They appreciate that it's user-friendly and it's free. And they can just hop on. You know, they're so tech savvy, all the kids. [School Stakeholder #3, Female]  But if their [youth] parents are not for mental health services. They can still download the app because they're all of age to consent, like for their own. [School Stakeholder #9, Female] |
|  | Referrals (n=10) | We live in a huge deficit area where we don't have a lot of providers, and kids who are having mental health issues either can't get care or don't want to seek care, because it takes too long. They may maybe don't like the providers that they've had in the past. I have provided it to multiple students so that they can access it quickly. [School Stakeholder #16, Female]  And during my assessments I get referrals from schools as well as from the community, and we determine together with the parents, whether or not the students could use additional support. And if that is the case, I will introduce them to Soluna. [Community Stakeholder #2, Female] |
|  | Stakeholder Presentation of Soluna (n=6) | Part of it is how you address it with them, because you can't coddle them. You can't make them feel like babies. You have to really just come at them like, 'Hey, you know. It can suck sometimes, being 16, you know a lot of times people don't hear you or you feel like you're not seen or heard. I get it.' And just staying away from certain words and terms helps. It's all in your approach, and it's all in how you deliver the information. [Community Stakeholder #12, Female]  It's just kind of opening their minds. And then I try really hard to tell them like, ‘this isn't a company that's trying to get you to do like a paid version of their app like the Calm app, unless you get the paid version you can't really access it, and like this is different than that like this is open to you.’ [School Stakeholder #9, Female] |
|  | Soluna Engagement Team (n=6) | I would be like 'have you tried Soluna?' And they'd say 'Oh, that's the guy right? The one with the purple shirt like.' 'Yes, that's him like, have you used the app?' 'Oh he said it in the classroom, but no, I haven't. Walk me through it.' So I think just that consistency allowed students to connect the person to the app, and knowing that it was something beneficial for them. [School Stakeholder #13, Female]  Knowing the way my community functions like a small town, so word of mouth is better than marketing. So I would think a representative and knowing just like they're gonna have to commute to where we are doing a presentation per district, where you could get all the ones who are doing something related to mental health, like academic counselors, social, emotional counselors. [Community Stakeholder #8, Female]  I feel like, if they were to like, have a representative go to a club meeting where they can talk about how to use the app in depth, how it can help students, or even if they did like a tabling event on campus for mental health awareness month, or something like that to be able to come and partner more with schools directly. [School Stakeholder #9, Female] |
|  | One-on-One Instruction (n=6) | Well, I think it's more like the one-on-one interactions. More so than maybe the classroom presentations, because…I know, like when I'm speaking to a student about like anxiety and stuff I'll have resources like physical handouts. But then also, I'll refer them to Soluna, and I'll be like, ‘Oh, like, there's some breathing exercises, there's journaling, and the chat.’ [School Stakeholder #15, Female]  The chances of them using the app without me kind of demoing or showing them the different features, I think, are much lower. [School Stakeholder #13, Female] |
|  | Hear from Other Youth (n=5) | I think it's just more ‘Oh, my friends, did this! Oh, it really helped me. Oh, I should try that kind of a thing.’ So getting kind of that build up of ‘Oh, I've had, like four friends access Soluna, and now I'm gonna try it, because it seems like they've had an easy time of it.’ [School Stakeholder #16, Female]  I think the biggest thing is word of mouth. Just like they hear it from me and they trust me. So then they're like, ‘okay, yeah, that sounds like a good resource.’ And then now at a point of hearing it from each other. [School Stakeholder #7, Female] |
| Barriers to Successful Real-World Implementation and Adoption | Lack of Instructions (n=13) | If you had like a demo where we could show all the counselors, like, look at all these things. And then when kids come in to see their counselor, they could access it, and you know, with the counselor, if they needed support. I like that. Because I feel like sometimes kids just don't like to jump off. They want someone to walk them through at one time. [School Stakeholder #16, Female]  I know there is also a feature to talk to someone. And I don't know much about that feature. So maybe kind of highlighting like ‘How does it really work? And who are the people you can really talk to?’ [School Stakeholder #14, Female]  It would be nice to have a testing version… I feel like we get students who are like, ‘oh, I tried the app,’ but they’re still like, ‘I'm not really sure what it's for or how to use it.’ So maybe the app having like ‘this is how you use it.’ Like little videos of quick videos on how to use Soluna cause I find that they're intrigued by getting a sticker or something, and then they try it and then they just leave it there because it's too complicated, or whatever. [School Stakeholder #11, Female] |
|  | Stakeholder Desire for Local Usage Data (n=9) | Many of our students take it, especially since it’s an app, and are like ‘Can I look at it, play with it’? But I don't know how many students use it on a regular basis and kind of stay with it. [School Stakeholder #14, Female]  But like the data of how many people have used it, how many students and if there's some sort of feedback that could be cool to share with students as well. [School Stakeholder #5, Female] |
|  | Phone Restrictions (Parental and School Policies) (n=8) | Their [phones] are not supposed to be out during the school day. So I think that might be one of the barriers is that they're not supposed to have it out, and they're supposed to be tucked in their backpack. [School Stakeholder #16, Female]  We have a newer, no cell phone policy this year at our school. So that makes it also hard to be like, 'get out your cell phone' because they're in a locked box on the teacher's desk during each class. I feel like that's been a little bit, maybe a small barrier, because it's not like I'm going into a class and holding up the poster and saying, scan this QR code because they don't have their phone. [School Stakeholder #5, Female]  Like sometimes students are grounded and they don't have their phones. [School Stakeholder #15, Female] |
|  | Lack of Awareness (n=7) | It's [Soluna] fairly new to us in the last couple of years. So I think we need more exposure to it. We just have to figure out how to make sure they know without interrupting their day, you know, during class time. [School Stakeholder #17, Female]  The hard part was getting them to understand like, it's okay you're not gonna be required to enter insurance information or payment information. This is just a personal app for you that you can use and just getting them to understand that. And then, once they did they saw the benefits of it. So if you can get past that initial hurdle of you know this is what this is for. [Community Stakeholder #12, Female] |
|  | Mental Health Stigma (n=5) | Well, I think maturity is one. The comfort level that they [youth] have with mental health. In some communities, there is a gigantic stigma attached to it. [Community Stakeholder #2, Female]  I do feel like in terms of the meditation aspect I feel like whenever I tell them about breathing and stuff that, they're always like, ‘that's weird.’ So I still feel like there's stigma. [School stakeholder #15, Female] |
